# A novel mRNA vaccine, SYS6006, against SARS-CoV-2

**DOI:** 10.3389/fimmu.2022.1051576

**Published:** 2023-01-05

**Authors:** Ke Xu, Wenwen Lei, Bin Kang, Hanyu Yang, Yajuan Wang, Yanli Lu, Lu Lv, Yufei Sun, Jing Zhang, Xiaolin Wang, Mengjie Yang, Mo Dan, Guizhen Wu

**Affiliations:** ^1^ National Institute for Viral Disease Control and Prevention, Chinese Center for Disease Control and Prevention, Beijing, China; ^2^ CSPC Pharmaceutical Group Co., Ltd., Shijiazhuang, Hebei, China; ^3^ State Key Laboratory of Novel Pharmaceutical Preparations and Excipients, CSPC Pharmaceutical Group Co., Ltd., Shijiazhuang, Hebei, China

**Keywords:** mRNA vaccine, SARS-CoV-2, Omicron variant, spike protein, neutralizing antibody, T cell immunity

## Abstract

The development of vaccines that can efficiently prevent the infection of SARS-CoV-2 is necessary to fight the COVID-19 epidemic. mRNA vaccine has been proven to induce strong humoral and cellular immunity against SARS-CoV-2. Here, we studied the immunogenicity and protection efficacy of a novel mRNA vaccine SYS6006. High expression of mRNA molecules in 293T cells was detected. The initial and boost immunization with a 21-day interval was determined as an optimal strategy for SYS6006. Two rounds of immunization with SYS6006 were able to induce the neutralizing antibodies against the SARS-CoV-2 wild-type (WT) strain, and Delta and Omicron BA.2 variants in mice or non-human primates (NHPs). A3^rd^ round of vaccination could further enhance the titers of neutralization against Delta and Omicron variants. *In vitro* ELISpot assay showed that SYS6006 could induce memory B cell and T cell immunities specifically against SARS-CoV-2 in mice. FACS analysis indicated that SYS6006 successfully induced SARS-CoV-2-specific activation of T follicular helper cell (Tfh) and Th1 cell, and did not induce CD4^+^Th2 response in NHPs. SYS6006 vaccine could significantly reduce the viral RNA loads and prevent lung lesions in Delta variant infected hACE2 transgenic mice. Therefore, SYS6006 could provide significant immune protection against SARS-CoV-2.

## Introduction

The coronavirus disease 2019 (COVID-19) is caused by severe acute respiratory syndrome coronavirus 2 (SARS-CoV-2). As of August 14, 2022, over 587 million confirmed cases and 6.4 million deaths had been reported worldwide (https://www.who.int/emergencies/diseases/novel-coronavirus-2019/situation-reports). There are currently five strains of SARS-CoV-2 listed by WHO as variants of concern (VOCs), including Alpha (B.1.1.7), Beta (B.1.351), Gamma (P.1), Delta (B.1.617.2) and Omicron (B.1.1.529) (1). The current global epidemiology of SARS-CoV-2 is characterized by the rapid spread of Omicron (2). Therefore, the development of vaccines that can efficiently prevent the infection of main variants of SARS-CoV-2, such as Delta and Omicron, will provide better options to fight the COVID-19 epidemic.

SARS-CoV-2 is a single-strand RNA virus and infects host cells through binding to a widely expressed receptor protein, angiotensin-converting enzyme 2 (ACE2), *via* its spike protein (S protein) (3). S protein is a type I transmembrane protein, including two subunits, S1 and S2. The receptor-binding domain (RBD) of S1 recognizes and binds to ACE2. S2 fuses the viral envelope with the host cell membrane to facilitate the entry of viral RNAs (4). Therefore, S protein is crucial in the process of SARS-CoV-2 infection. S protein or RBD domain is a common target for the development of SARS-CoV-2 vaccines (5).

At present, the two mRNA vaccines that have been approved for marketing or emergency use are Spikevax, developed by Moderna and Comirnaty, developed by Pfizer/BioNTech (6, 7). The vaccines enter the host cells by endocytosis, then their mRNAs are released from lipid nanoparticle (LNP) and express S antigens by the ribosome. The S antigens induce immune responses mainly in two distinct ways. The intracellular antigens are digested into polypeptides and presented to cytotoxic T cells by MHC class I complex. Subsequently, the activated cytotoxic T cells directly secret perforin and granzyme to induce the apoptosis of infected cells. The extracellular antigens can be taken up and broken down into polypeptides by antigen-presenting cells and presented to helper T cells through MHC class II complex. The activated helper T cells stimulate B cells to produce antibodies and induce macrophages to eliminate pathogens through inflammatory cytokines (8). Therefore, mRNA vaccines can induce the clearance of infected SARS-CoV-2 in host through the dual mechanisms of both cellular and humoral immunities. Results from clinical trials of the two mRNA vaccines, Comirnaty and Spikevax, showed that their protective efficacies against SARS-CoV-2 infection were more than 90% after the two-dose vaccinations. The most common adverse effects included headache, joint pain, muscle pain, fatigue, fever, pain and swelling at the injection site, which were entirely tolerable (6, 7). Therefore, mRNA vaccines show controllable safety and high efficacy in the control of COVID-19.

SYS6006 is a SARS-CoV-2 mRNA vaccine developed by Zhongqi Pharmaceutical Technology Co., Ltd. of CSPC Pharmaceutical Group, which combines mRNA molecules with lipids to form lipid nanoparticles. The mRNA molecules were designed according to the S protein sequence of the prototype SARS-CoV-2 strain and the key mutations of main epidemic variants. To enhance immunogenicity, K986P and V987P mutations (S-2P) are also introduced to maintain prefusion conformation of the encoded antigen (9). Here, we demonstrated that SYS6006 was able to induce the neutralizing antibodies against the SARS-CoV-2 wild-type (WT) strain, and Delta and Omicron BA.2 variants in mice or non-human primates (NHPs). SYS6006 could successfully induce SARS-CoV-2-specific memory B cell immunity, the activation of T follicular helper cells (Tfh) and Th1 cells, and did not induce CD4^+^Th2 response in NHPs. Furthermore, SYS6006 vaccine could significantly reduce the viral RNA loads and completely prevent lung lesions induced by Delta variant infection in hACE2 transgenic mice. The significant immune protection against SARS-CoV-2 supports further clinical development of SYS6006 in humans.

## Materials and methods

### Mice

Pharmaron TSP was fully credited by the International Experimental Animal Assessment Committee (AAALAC). All procedures utilized in the study were in accordance with “Guide for the Care and Use of Laboratory Animals” and Pharmaron TSP Institutional Animal Care and Use Committee (IACUC) policies. An animal care and use application for this study was reviewed and approved by Pharmaron TSP IACUC (IACUC No.: 21-375).

#### Non-human primates

Certified commercial monkey diet of approximately 200 g/day (Beijing Keaoxieli Feed Co., Ltd., SCXK (Jing) 2019-0003, Lot No.: 21068211, 21078211, 21088211, 21088221, 21098211) was provided twice daily and fruits of approximately 50 g/day once daily to each monkey, except for scheduled fasting times. Nutritional ingredients (tested for moisture, protein, fat, fiber, ash, calcium, and phosphorus), chemical parameters (tested for arsenic, lead, mercury, cadmium, benzene hexachloride, clofenotane, and aflatoxin B1), and microbiological testing (aerobic plate count, coliform, mold and yeasts count, and pathogenic bacteria (Salmonella)) in the diet of every batch were analyzed by Pony Testing Technology Tianjin Co., Ltd. and Centre Testing International (Qingdao) Co., Ltd. The diet met the State Standard of the People’s Republic of China GB14924.2-2001 and GB14924.3-2010. Animal care and management were compliant with the relevant Standard Operating Procedures (SOPs) of JOINN Laboratories, the Guide for the Care and Use of Laboratory Animals, 8th Edition (Institute of Laboratory Animal Resources, Commission on Life Sciences, National Research Council; National Academy Press; Washington, D.C., 2010), and the U.S. Department of Agriculture (USDA) through the Animal Welfare Act (Public Law 99-198). JOINN Laboratories is fully accredited by the Association for Assessment and Accreditation of Laboratory Animal Care International (AAALAC). Procedures used in this study were approved by the Institutional Animal Care and Use Committee (IACUC) at JOINN Laboratories (IACUC serial number: ACU21-1700).

### Cells, viruses and proteins

African green monkey kidney cells Vero (ATCC, CCL-81) and human embryonic kidney cells HEK 293T (ATCC, CRL-11268) were maintained in Dulbecco’s minimal essential medium (DMEM; Thermo Fisher Scientific) supplemented with 10% fetal bovine serum (FBS; Thermo Fisher Scientific) and penicillin (100 U/mL)-streptomycin (100 mg/mL) (Thermo Fisher Scientific). Patient-derived SARS-CoV-2 isolates, including wild-type, Delta and Omicron variants, were passaged in Vero cells, and the virus stock was aliquoted and titrated according to the tissue culture infective dose 50% (TCID50) in Vero cells (10). All experiments on SARS-CoV-2 infection were performed under Biosafety Level 3 facilities. The recombinant RBD protein, the peptide pools of S protein and the entire S protein were respectively obtained from GenScript and WuXi AppTec.

### mRNA synthesis

The mRNA was produced *in vitro* using T7 RNA polymerase-mediated transcription from a linearized DNA template, which encodes full length spike protein of SARS-CoV-2 carrying the key mutations of major epidemic strains. Besides that, the 5’ and 3’ untranslated regions and a poly-A tail were also incorporated into the synthesized mRNA.

### Lipid-nanoparticle encapsulation of the mRNA

Lipids were dissolved in ethanol, and the mixture contains ionizable lipids, DSPC, cholesterol, etc. The lipid mixture was then combined with 20 mM citrate buffer (pH4.0) containing mRNA at a ratio of 1:2 through a designed micro-channel device. After that, formulations were then diafiltrated against the 10×volume of PBS (pH7.4) through a tangential flow filtration (TFF) membrane with a cut-off of 140 kD molecular weight. Subsequently, the mRNA nanoparticles were concentrated to desired concentrations, passed through a 0.22 μm filter, and stored at 2-8°C until use. All formulations were tested for particle size, particle distribution, RNA concentration and encapsulation.

### mRNA transfection

HEK293T cells were seeded in 6-well plates at 600,000 cells/well. Eighteen hours later, the cells were transfected with 4 μg mRNA of S protein using Lipofectamine 2000 Transfection Reagent (Thermo Fisher Scientific). Cells were collected 24 hours after transfection. The expression of mRNA molecules was then detected by western blot and flow cytometry analysis (FACS) with MonoRab™ SARS-CoV-2 Neutralizing Antibody (4G6) (Genscript).

### Mouse immunization

Ten of 6-8-week-old female BALB/c mice were immunized intramuscularly with various doses of SYS6006 and boosted with equal dose on day 14, 21or 28 post initial immunization. Sera were collected at day 7 after boost immunization for detection of SARS-CoV-2-specific IgG and neutralizing antibodies. Spleen tissues were collected at day 28 post initial immunization for evaluation of cellular immune responses.

### Evaluation of sera antibody titers

S1-specific IgG antibody titers against Delta variant were determined by ELISA assay. Briefly, 10-fold serial dilutions of the inactivated serum starting at 1:10000, were added into the 96-well plates (100 μL/well) coated with the recombinant Delta S1 antigen. The plates were incubated at 37°C for 60 minutes. After washing three times with wash buffer, the plates were added with Horseradish peroxidase (HRP)-conjugated goat anti-mouse IgG (1:10000). Then, the plates were washed five times with wash buffer and added with TMB substrate, followed by 10 minutes of incubation at room temperature. After that, the absorbance (450 nm) was read using a microplate reader.

Neutralizing antibody titers against WT strain, and Delta and Omicron variants of SARS-CoV-2, respectively, were determined using the corresponding live virus-based neutralization assay. 2-fold serial dilutions of the serum, starting at 1:40, were mixed with 100 TCID50 live viruses in a 1:1 (vol/vol) ratio, followed by incubation at 37°C for 2 hours. After that, Vero cells were incubated with the serum-virus mixtures at 37°C for 2 days, and the cytopathic effects (CPE) of vero cells were detected by CellTiter-Glo Luminescent Cell Viability Assay (11). The 50% neutralization titer (NT50) was calculated according to the antibody titer in serum with 50% inhibition of CPE.

### Cynomolgus monkey immunization studies

A total of 20 adult cynomolgus monkeys with similar age and weight were randomly assigned into two groups. The animals in these two groups were immunized intramuscularly with 30 μg and 90 μg of mRNA vaccine, respectively, and boosted twice with a 21-day interval using the same dose. Blood was collected on day 35 and day 50 after initial immunization to detect neutralizing antibodies.

### Enzyme-linked immunospot assay

The immune responses elicited by the designed mRNA vaccine in the vaccinated mice were assessed using mouse IgG and IFN-γ ELISpot kits (Mabtech), according to the manufacturer’s protocol. Briefly, the plates were blocked using RPMI 1640 (Thermo Fisher Scientific) containing 10% FBS and incubated for 30 minutes. Splenocytes from immunized mice were then plated at 300,000 cells/well with RBD, S protein, or peptide pool for SARS-CoV-2. After incubation at 37°C for 36 hours, the plates were washed with wash buffer, and biotinylated anti-mouse IgG or IFN-γ was added into each well, followed by incubation for 2 hours at room temperature. After the addition of chromogenic substrate for 6.5 minutes at room temperature, the reaction was terminated by washing under running water for 2 minutes, and the plates were dried at 37°C for 30 minutes. Finally, the spots were counted using an ELISpot counter (AID).

### Mouse infection experiments

The hACE2 transgenic mice were supplied by National Institutes for Food and Drug Control. Two groups of 6-8-week-old female hACE2 transgenic mice were immunized intramuscularly with SYS6006 mRNA-LNP (5 μg/dose, n = 9; 20 μg/dose, n = 9), and boosted with equal dose on day 21 after the initial immunization. Sera were collected starting on day 7 after the initial immunization with a 7-day interval to detect the S1-specific IgG against Delta. The immunized mice were infected intranasally with the live virus of Delta variant (5×10^4.33^ TCID50/mouse) on day 12 after boost immunization. 7 days after the infection, all animals were sacrificed, and the lung tissues were collected for the subsequent viral RNA detection and histopathology assay.

### Quantification of viral RNA in the lung tissue of the infected mouse by RT-qPCR

Viral RNA in the lung tissues from the infected mice was detected by quantitative reverse transcription PCR (RT-qPCR). Briefly, tissue samples were weighed and homogenized. Viral RNA in the tissues was extracted using the QIAamp Viral RNA Mini Kit (QIAGEN) according to the manufacturer’s protocol. The viral RNA quantification was performed by RT-qPCR targeting ORF1ab and N genes of SARS-CoV-2 using One Step PrimeScript RT-PCR Kit (Takara). The detected viral RNA loads were expressed on a log10 scale after comparison with a standard curve produced using the ten-fold serial dilutions of SARS-CoV-2 RNA.

### Histopathology assay

For histopathologic examination, lung tissues from the infected mice were fixed in 4% neutral-buffered formaldehyde for 48 hours, which were then embedded in paraffin, sectioned, and stained with hematoxylin and eosin (H&E). Tissue sections were examined microscopically by a pathologist. Original magnification of the observation was set to 20.

### Statistical analysis

Data were analyzed using GraphPad Prism (GraphPad Software, San Diego, CA, USA). ANOVA analysis, Mann-Whitney or t-test was used to determine the statistical significance among different groups.

## Results

### The expression of mRNA molecules as a vaccine

**Figure 1:**
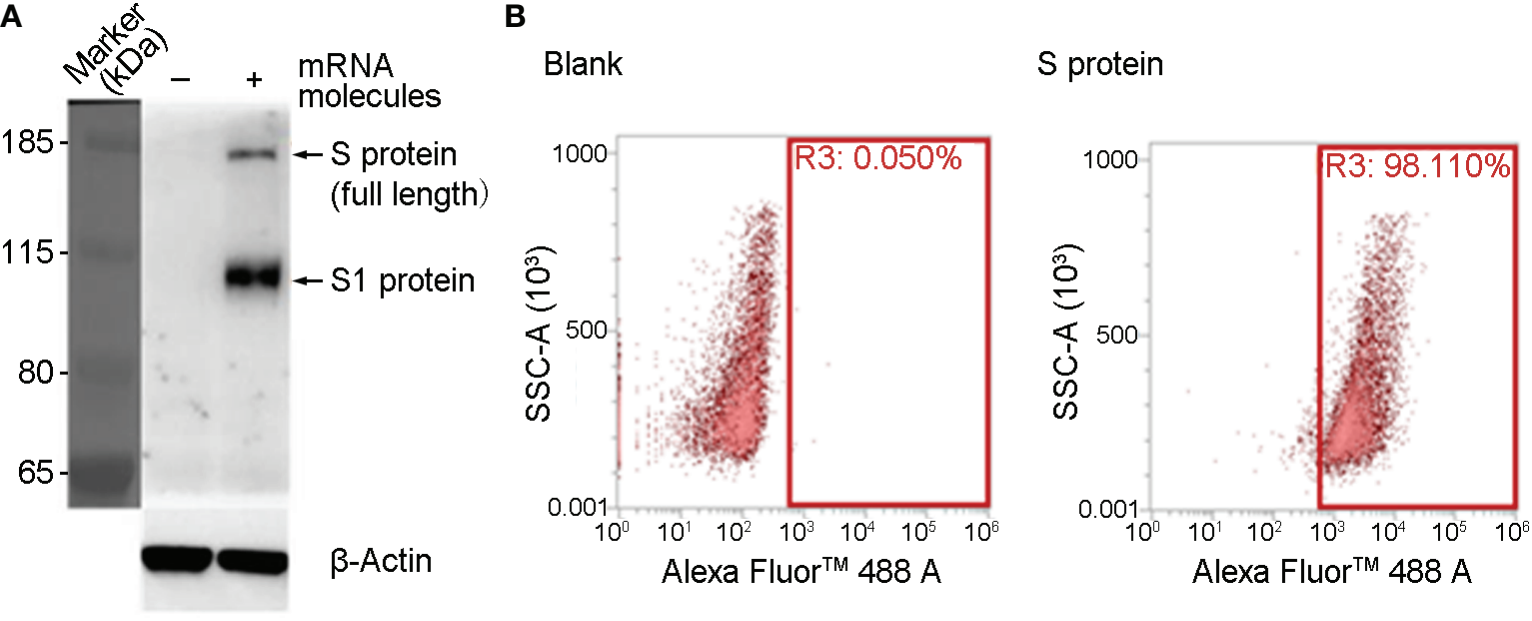
The expression of SYS600 mRNA molecule in 293T cells. 293T cells were transfected with mRNA molecules of SYS6006 for 24 hours. The expression of S protein was detected respectively by western blot **(A)** and FACS analysis **(B)**.

SYS6006 is a SARS-CoV-2 mRNA vaccine combining mRNA molecules with lipids to form lipid nanoparticles. The mRNA molecules were designed according to the S protein sequence of the prototype SARS-CoV-2 strain, and contained several key mutation sites present in pandemic strains including Delta, BA.4, BA.5, and BF.7. The mutations of K986P and V987P were also introduced in SYS6006 to maintain the prefusion form of spike protein, which could induce more specific neutralizing antibodies when expressing *in vivo*, and were also included in Pfizer-BioNTech (BNT162b2, Comirnaty) and Moderna (mRNA-1273, Spikevax) mRNA vaccines (12, 13). To validate the immunogenicity and protection of SYS6006 against SARS-CoV-2, we first detected the expression of mRNA molecules in 293T cells. The full-length S protein could be detected by western blot 24 hours after mRNA transfection (**Figure 1A**). The results of FACS showed that the ratio of cells with S protein expression is 98.11% (**Figure 1B**). Therefore, the mRNA molecules in SYS6006 can be translated into target antigen, S protein, in human cells.

### SARS-CoV-2-specific antibodies induced by SYS6006 under different immunization strategies

**Figure 2:**
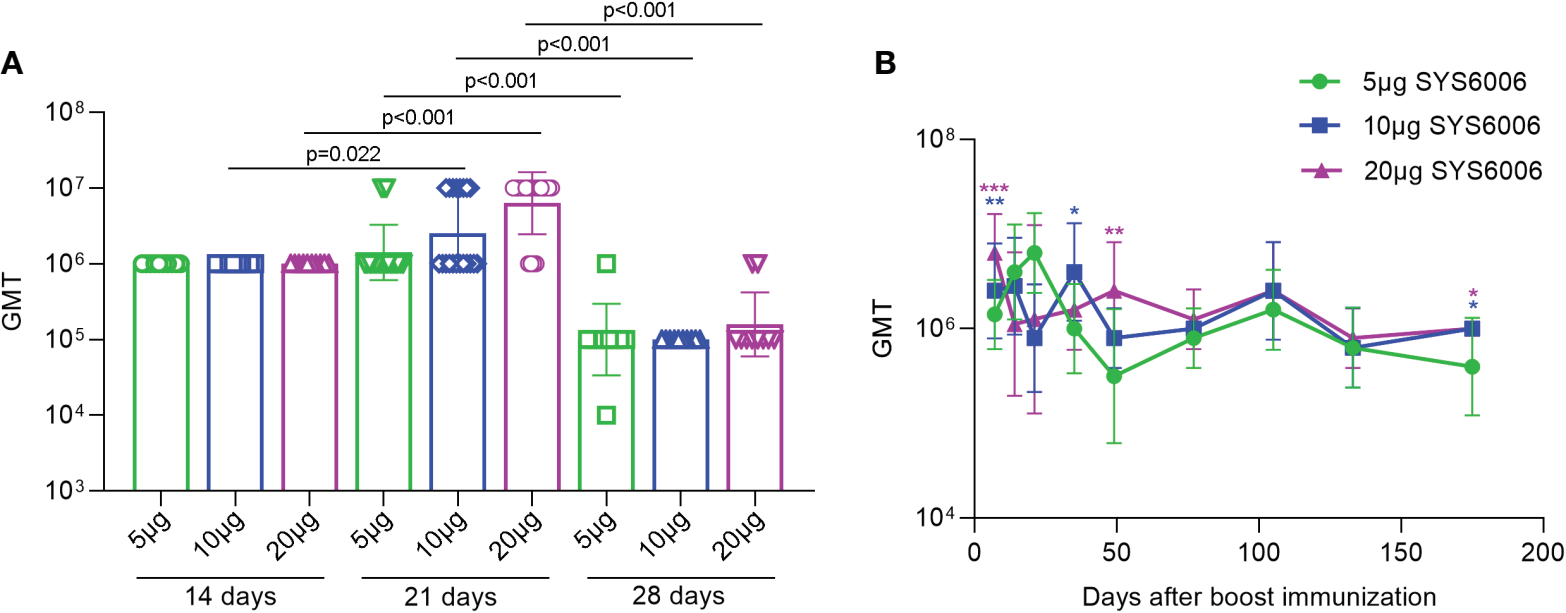
The GMTs of S1-specific IgG antibodies against Delta variant in the sera of the vaccinated mice under various immunization strategies. 6-8-week-old female BALB/c mice were immunized intramuscularly with the indicated doses of SYS6006 and boosted with an equal dose on day 14, 21 or 28 post initial immunization. Sera were collected from mice 7 days after boost immunization and used to detect the geometric mean titers (GMTs) of S1-specific IgG antibodies against Delta variant **(A)**. Sera were also collected from mice at the indicated time points after 2-dose immunization with 21-day interval and used to detect the GMTs of S1-specific IgG antibodies against Delta variant **(B)**. Data are presented as geometric mean ± geometric SD. *P<0.05, **P<0.01, ***P<0.001 vs. mice immunized with 5μg of SYS6006.

To determine the optimal immunization strategy for SYS6006, BALB/c mice were immunized intramuscularly with 5, 10 or 20 μg of mRNA vaccine, respectively, and then boosted with another dose on day 14, 21 or 28. The sera were collected from the immunized mice 7 days after boost vaccination and the IgG antibodies in the sera were measured using ELISA assay. The geometric mean titers (GMTs) of the S1-specific antibodies against Delta variant were over 1:10^5.0 for all the tested immunization strategies with different prime-boost intervals. The boost immunization on day 21 induced the highest GMT among these 3 strategies (**Figure 2A**). The S1-specific antibody GMT against Delta variant was still over 1:10^6 175 days after the boost immunization for the immunization strategy with a 21-day interval. The S1 antibody GMTs under the immunization of 10 and 20μg SYS6006 were significantly higher than that of 5μg SYS6006 (**Figure 2B**). Moreover the S1 antibody GMT against Delta variant in monkeys received 30μg SYS6006 twice with a 21-day interval maintained at 1:10^4 190 days after the first immunization (**Figure S1**). These results indicated the persistence of immune protection by SYS6006. Therefore, the immunization scheme of two-dose vaccination with a 21-day interval was optimal and selected for further studies.

### Neutralizing antibodies induced by SYS6006 in mice and NHPs

**Figure 3:**
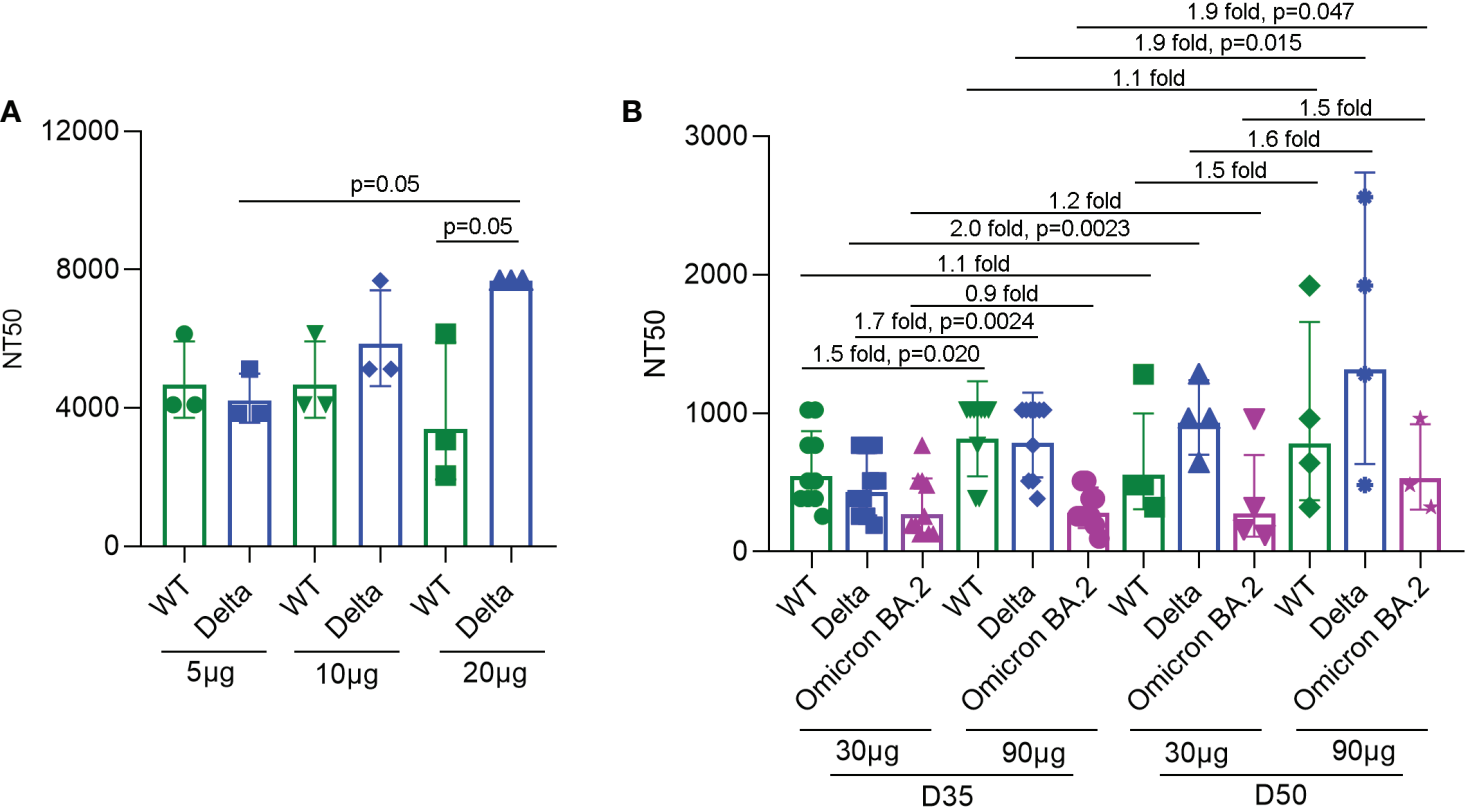
NT50s of neutralizing antibodies against the SARS-CoV-2 WT strain, as well as Delta and Omicron BA.2 variants under SYS6006 immunization. **(A)** 6-8-week-old female BALB/c mice were immunized intramuscularly with the indicated doses of SYS6006 and boosted with the same dose on day 21 post initial immunization. Sera were collected from mice 14 days after boost immunization. The serum samples from 3 or 4 mice were mixed and used to test NT50s of neutralizing antibodies against SARS-CoV-2 wild-type strain and Delta variant. **(B)** Cynomolgus macaques were intramuscularly immunized with the indicated doses of SYS6006 and boosted twice with a 21-day interval using the same dose. The sera were collected 35 days (D35) and 50 days (D50) after the initial vaccination. The NT50s of neutralizing antibodies in sera against SARS-CoV-2 WT strain, and Delta and Omicron BA.2 variants were determined. Data are presented as geometric mean ± geometric SD. NT50, neutralization titer with 50% inhibition of cytopathic effect, WT, wild-type. The fold changes and statistical significances between NT50s of corresponding groups were presented on the columns.

To detect the neutralizing antibody levels against SARS-CoV-2 virus induced by SYS6006, ten BALB/c mice were immunized intramuscularly with 5, 10, or 20 μg of the designed mRNA vaccine and boosted with the same dose on day 21. The sera were collected from the immunized mice 14 days after boost vaccination and incubated with the wild-type SARS-CoV-2 as well as the Delta variant. Then the serum-virus mixtures were incubated with Vero cells to detect the cytopathic effect. According to the neutralization titers of antibodies in sera with 50% inhibition of CPE, SYS6006 was able to induce the production of neutralizing antibodies against both WT strain and Delta variant. The neutralization against Delta variant induced by 20 μg dose was significantly higher than that induced by 5 μg dose (**Figure 3A**).

To detect the neutralization capability of SYS6006 in NHPs, cynomolgus macaques were intramuscularly immunized with mRNA vaccine and boosted twice with a 21-day interval using the same dose. The sera were collected 14 days after the 2^nd^ vaccination (D35) and 8 days after the 3^rd^ vaccination (D50). The neutralization titers of sera against the WT strain, and Delta and Omicron BA.2 variants were determined according to the 50% inhibition of CPE in Vero cells infected with the mixture of serum and virus. As shown in **Figure 3B**, both the immunization schemes with different doses (30 and 90 μg) of SYS6006 were able to induce the neutralization antibodies against the WT strain, as well as Delta and Omicron variants. The NT50s of antibodies against WT and Delta for the dose of 90 μg were significantly higher than those of 30 μg dose on D35. In addition, compared with two-dose vaccination, three-dose vaccination could further enhance the neutralization capability against Delta variant under both 30 and 90 μg immunization, and also enhance the neutralization capability against Omicron variant under 90 μg immunization.

### The activity of memory B cells induced by SYS6006 in mice

**Figure 4:**
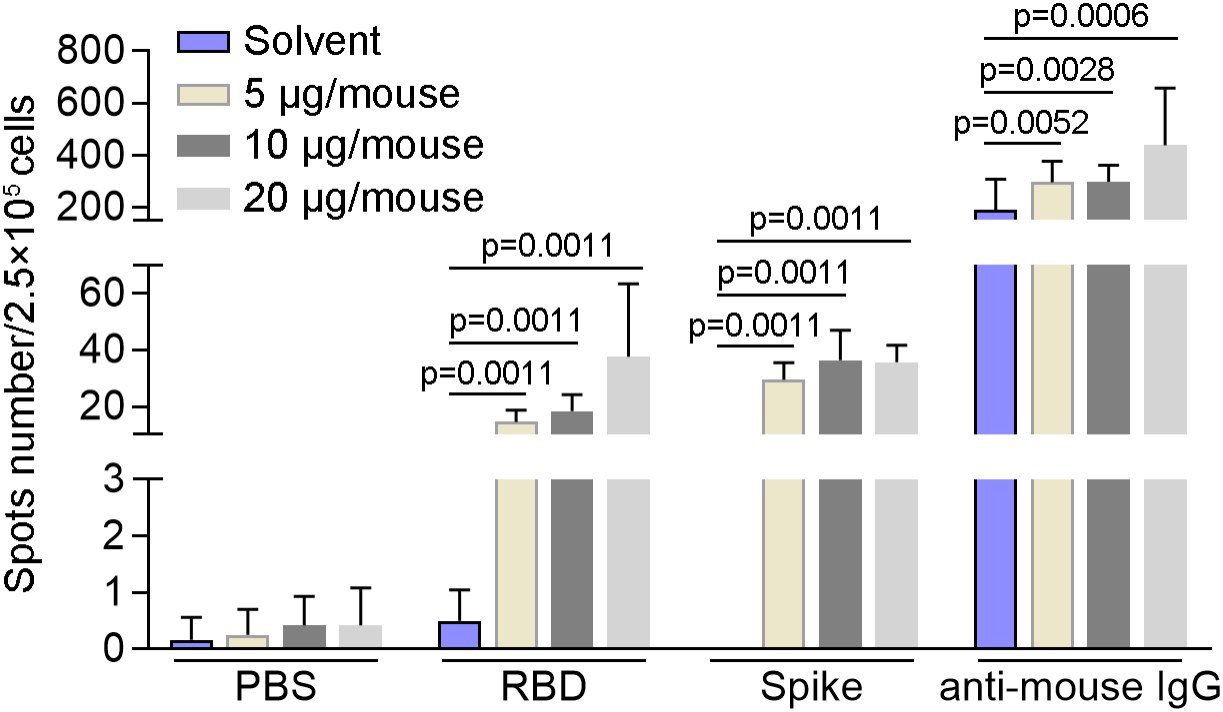
The specific activity of B cells induced by SYS6006 in mice. 6-8-week-old female BALB/c mice were immunized intramuscularly twice with the indicated doses of SYS6006 with a 21-day interval. The splenocytes were collected 7 days after the 2^nd^ vaccination, stimulated with RBD and spike antigens, and tested for B cell activity *via* mouse IgG ELISpot assay (n=6). Data are presented as mean ± SD. RBD, receptor-binding domain.

In order to detect the activity of memory B cells induced by SYS6006, BALB/c mice were intramuscularly immunized with 5, 10, or 20 μg of mRNA vaccine and boosted with the same dose on day 21. The splenocytes were collected 7 days after boost vaccination and tested for B cell activity *via* mouse IgG ELISpot assay. As shown in **Figure 4**, all the three immunization schemes with different doses induced B cells specifically recognizing RBD and S proteins of SARS-CoV-2. After *in vitro* expansion with R848 and IL2 for 4 days, B cells in all the mice recognized RBD and spike proteins of SARS-CoV-2, suggesting that SYS6006 could induce memory B cells specifically against SARS-CoV-2 in mice (**Figure S2A, B**).

### The T cell immunity against SARS-CoV-2 induced by SYS6006 in mice and NHPs

**Figure 5:**
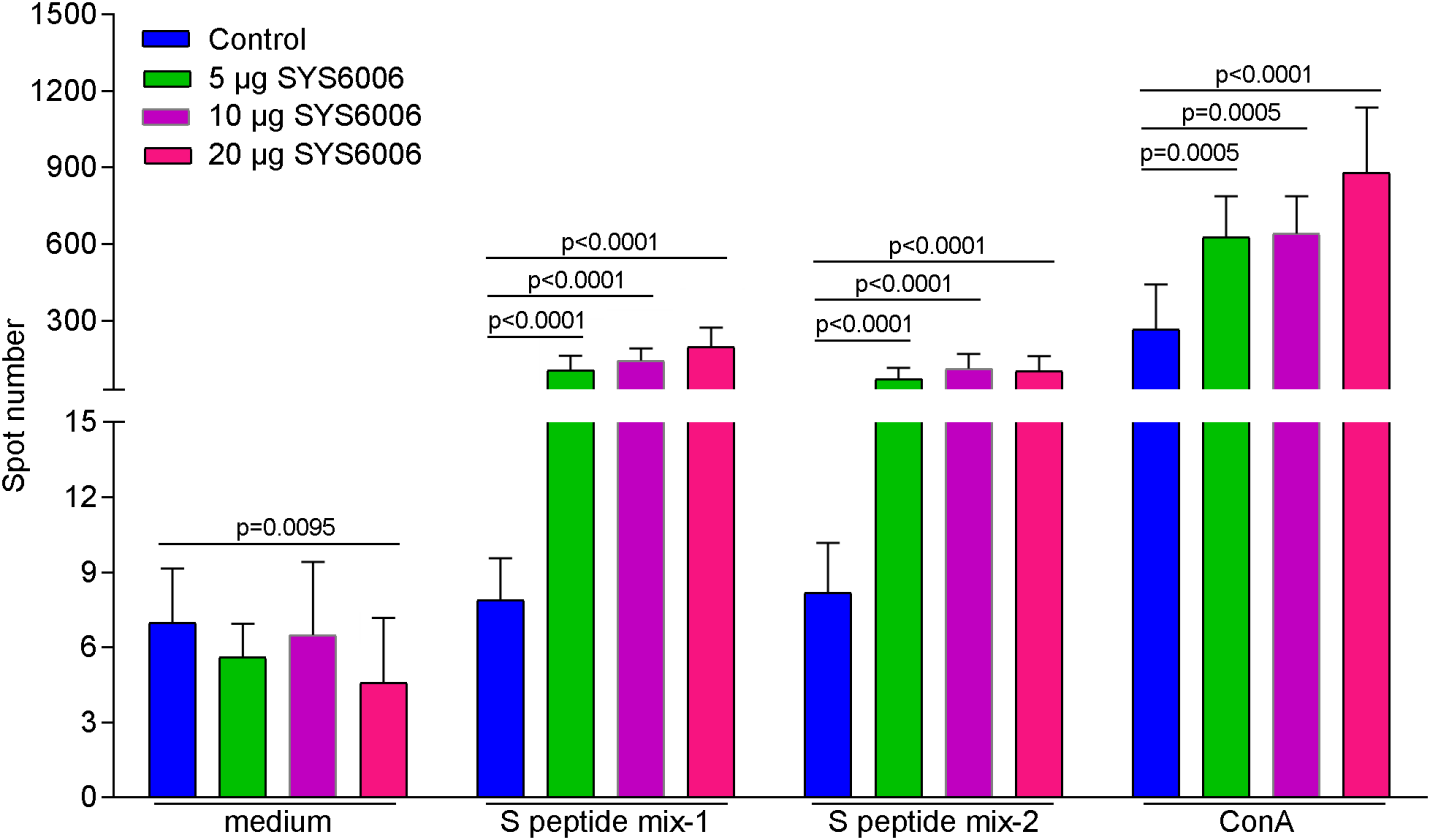
The T cell immunity against SARS-CoV-2 induced by SYS6006 in mice. 6-8-week-old female BALB/c mice were immunized intramuscularly with the indicated doses of SYS6006 and boosted with the same dose on day 21 post initial immunization. The splenocytes were collected 14 days after boost vaccination, stimulated with 2 peptide mixtures of S protein, and tested for T cell activity *via* mouse IFN-γ ELISpot assay (n=10). Data are presented as mean ± SD. Concanavalin A (ConA) was positive control to stimulate T cells.

In order to detect the T cell immunity induced by SYS6006, BALB/c mice were intramuscularly immunized with 5, 10 or 20 μg of mRNA vaccine and boosted with the same dose on day 21. The splenocytes were collected 14 days after boost vaccination, and stimulated with 2 peptide pools of SARS-CoV-2 S protein. Then the T cell immunity was detected *via* mouse IFN-γ ELISpot assay. As shown in **Figure 5**, all the three immunization schemes with different doses induced significant T cell response specifically against S protein of SARS-CoV-2.

**Figure 6:**
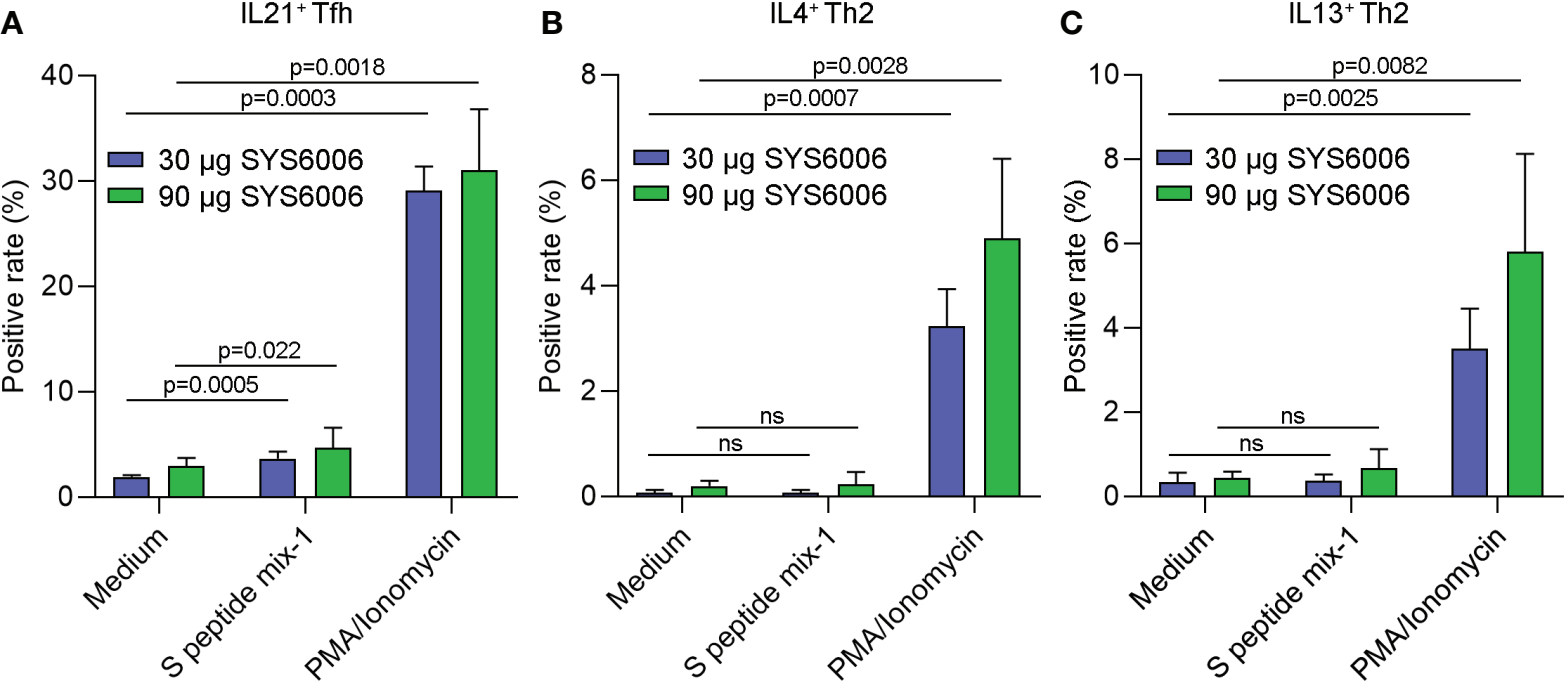
The antigen-specific Tfh and Th2 cells in NHPs immunized with two different doses of SYS6006. Cynomolgus macaques were intramuscularly immunized with the indicated doses of SYS6006 and boosted with a 21-day interval using the same dose. The peripheral blood mononuclear cells were collected from NHPs 50 days after the 1^st^ vaccination, stimulated with a peptide pool of SARS-CoV-2 S protein, and tested for the rates of IL21^+^ Tfh cells **(A)**, and IL4^+^ **(B)** and IL13^+^ **(C)** Th2 cells *via* FACS analysis (n=3). Data are presented as mean ± SD. PMA/Ionomycin was positive control to stimulate T cells. ns, no significance; NHPs, non-human primates; Tfh, T follicular helper cell; PMA, phorbol 12-myristate 13-acetate.

To further detect the T cell immunity induced by SYS6006 in NHP, cynomolgus monkeys were intramuscularly immunized with 30 or 90 μg of mRNA vaccine and boosted with the same dose on Day 21. The peripheral blood mononuclear cells were collected 50 days after primary vaccination, and stimulated with a peptide pool of SARS-CoV-2 S protein. Then the T cell immunity was detected *via* FACS analysis. As shown in **Figure 6A**, the activation of T follicular helper cell (CD4^+^ CCR7^+^CD45RA^−^CXCR5^+^ICOS^+^Tfh) against S protein was enhanced in NHPs immunized with both lower and higher doses of SYS6006.The gating strategy for IL21^+^ Tfh cell was presented in **Figure S3** (14–17). The SARS-CoV-2-specific CD4^+^Th1 and Th2 cells were also detected by FACS analysis. The gating strategies for Th1 and Th2 cells were presented on **Figure S4**. The ratios of IL2^+^ and IFNγ^+^ Th1 cells against S protein were enhanced in NHPs immunized with both lower and higher doses of SYS6006 (**Figure S5A, B**). The Th2 cells were not affected under both lower and higher doses of SYS6006 as shown by intracellular staining of IL4 and IL13 (**Figures 6B, C**). Therefore, our results demonstrated that the mRNA vaccine SYS6006 successfully induced SARS-CoV-2-specific Tfh and Th1 activation, which supported durable B and T cell immune responses. The Th2 response was not affected by SYS6006, which indicated the safety of this mRNA vaccine.

### SYS6006 vaccine protected hACE2 mice from lung lesions induced by the Delta variant

To further evaluate the protective efficacy of SYS6006 *in vivo*, female hACE2 mice were immunized intramuscularly with 5 or 20 μg of mRNA vaccine and boosted with the same dose on day 21. Live-virus infection of Delta variant was conducted *via* intranasal instillation in the vaccinated and control mice on day 12 after boost vaccination. The amount of Delta variant is 5×10^4.33^ TCID50 per mouse. The GMTs of S1-specific IgG antibodies against Delta variant were kept at 1:10^6 both in 5 and 20 μg groups during the vaccination and virus infection processes.

**Figure 7:**
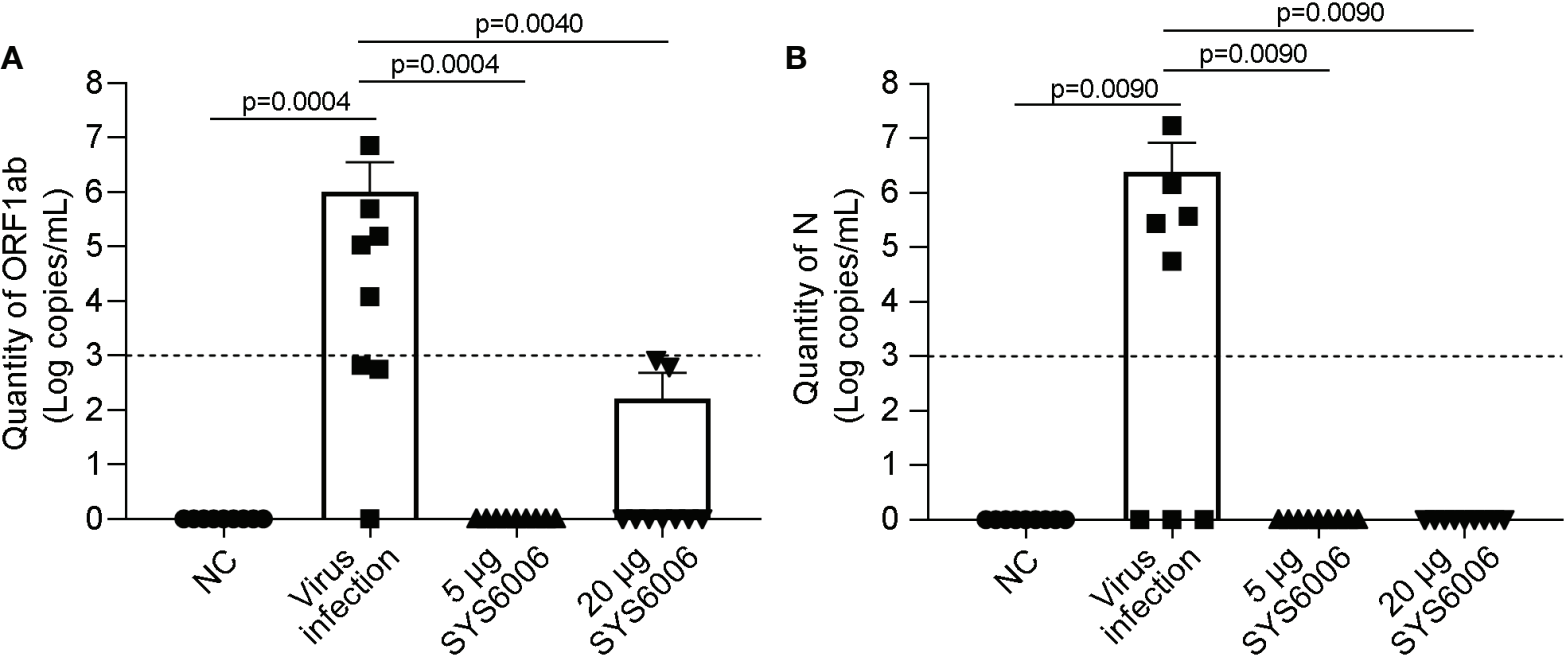
The viral loads in the lung tissues of hACE2 transgenic mice under the infection with Delta variant. 6-8-week-old female hACE2 transgenic mice were immunized intramuscularly with SYS6006, boosted with the same dose on day 21 after the initial immunization, and intranasally infected with live-virus of Delta variant on day 12 after boost vaccination. Lung tissues were collected 7 days after virus infection and tested for the quantities of ORF1ab **(A)** and N **(B)** RNAs by RT-qPCR. NC means the control mice without immunization and virus infection. “Virus infection” group represents the mice did not receive vaccination but were infected by live virus. “5 μg SYS6006” and “20 μg SYS6006” groups mean the mice were vaccinated with 5 μg and 20 μg of SYS6006 respectively, and then infected by live virus. The dot line indicated the detection limit of Real-time PCR (1000 copies/mL virus RNA and cycling threshold (CT) =35). Data are presented as mean ± SD. ACE2, angiotensin-converting enzyme 2.

The mice were sacrificed 7 days after live-virus infection with the Delta variant. The copies of ORF1ab and N genes in the lung tissues of the infected mice were detected *via* RT-qPCR to determine viral RNA loads, and histopathological examination of the lung tissues was also performed. No obvious viral RNAs were detected in the lungs of the mice both in the 5 and 20 μg vaccination groups. In contrast, high levels of viral RNAs were detected in the lungs of the infected ed mice in the control group, with 10^6^and 10^6.38^ copies/mL for ORF1ab and N genes, respectively (**Figures 7A, B**). Thus, both the immunization schemes with the dose of 5 and 20 μg could significantly reduce the viral RNA loads of the Delta variant.

**Figure 8:**
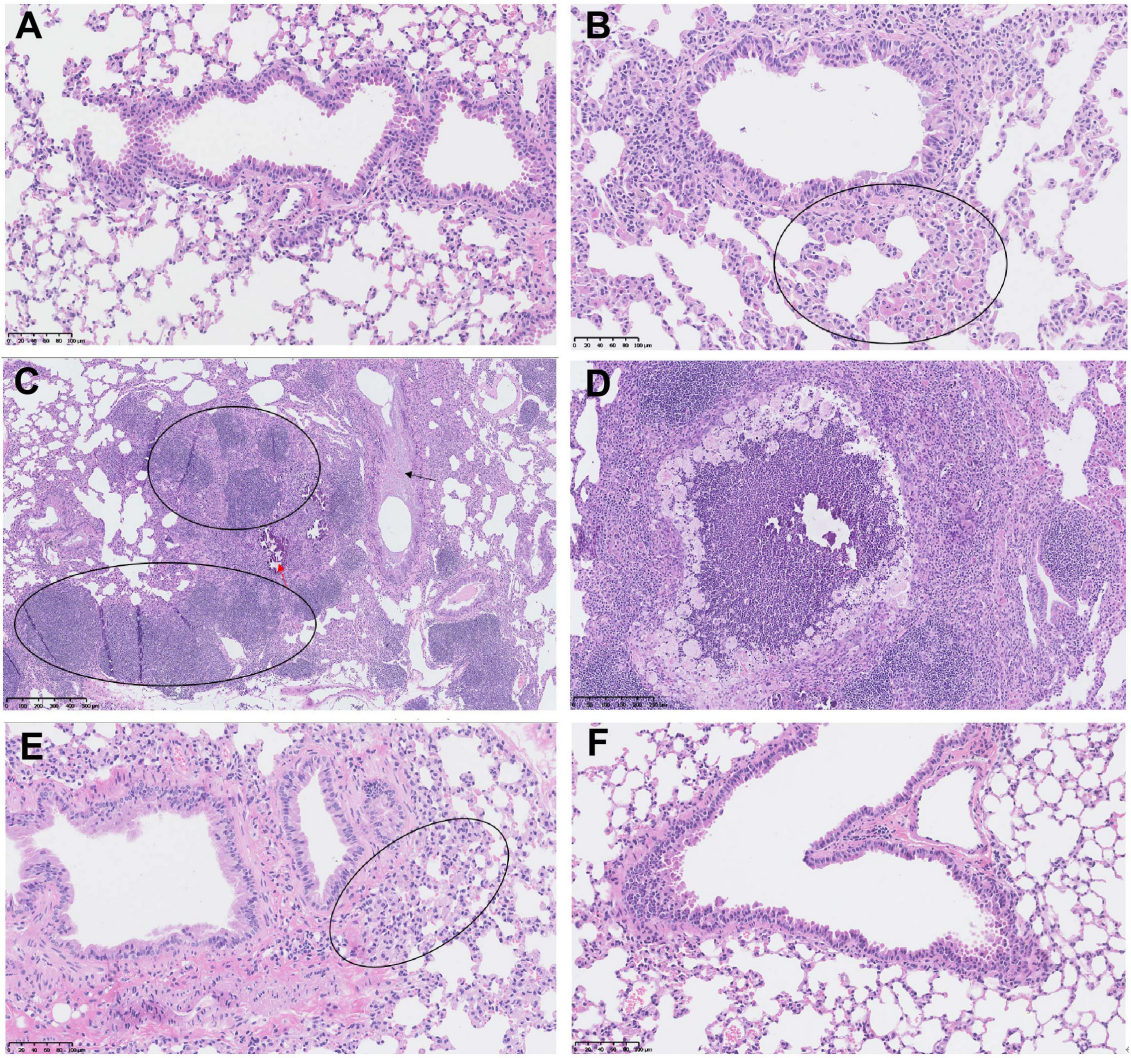
The lung lesions in the hACE2 transgenic mice under the infection with Delta variant. **(A)** The lung tissue of the control mouse without SYS6006 immunization and virus infection (20×); **(B-D)** The lung tissues of the mice without SYS6006 immunization infected by Delta virus. The marked circles and arrows in the figures indicate moderate macrophage aggregation (circle in B, 20×), moderate lymphocyte proliferation (circle in C, 5×), mild thickened alveolar septa (black arrow in C, 5×), slight mineralization (red arrow in C, 5×), and regional necrosis (D, 10×), respectively; **(E)** The lung tissue of the mouse infected by Delta virus after the immunization with 5 μg of SYS6006 (20×). The circled region indicates slight macrophage aggregation; **(F)** The lung tissue of the mouse infected by Delta virus after the immunization with 20 μg of SYS6006 (20×).

More importantly, the control mice developed typical lung lesions, including macrophage aggregation (minimal to moderate, 5 out of 8 mice), lymphocyte proliferation (moderate, 1 out of 8), pulmonary mineralization (minimal, 1 out of 8), mucus in bronchiole (minimal, 1 out of 8), thickened alveolar septa (mild, 1 out of 8), and regional necrosis (mild, 1 out of 8). Whereas, only 1 out of 9 mice in the 5μg-dose vaccination group developed macrophage aggregation (minimal), and no lung lesion was detected in all 9 mice in the 20μg-dose group (**Figure 8**). These results demonstrated that two doses of SYS6006 vaccine with 20 μg/dose completely protected mice from lung lesions induced by the Delta variant.

## Discussion

Previous reports indicated that mRNA vaccine could induce both cellular and humoral immunities to block SARS-CoV-2 infection (8). The present study demonstrated that 2- or 3-dose immunization of SYS6006 was able to induce the neutralizing antibodies against the WT SARS-CoV-2 strain, and the Delta and Omicron variants in mice and NHPs. The 3^rd^ dose of vaccination could further enhance the titers of neutralization against Delta and Omicron variants. ELISpot and FACS analysis showed that SYS6006 could induce SARS-CoV-2-specific memory B cell and T cell immunities in mice, and Tfh and Th1 activation in NHPs. And the vaccine did not induce Th2 immune response. Twenty micrograms of SYS6006 vaccine could significantly reduce the viral RNA loads of Delta variant in the lung tissue, and completely prevent the lung histopathology induced by Delta variant infection in hACE2 transgenic mice. Therefore, SYS6006 could elicit significant humoral and cellular immune responses against SARS-CoV-2.

The neutralization titers against Omicron BA.2 were down-regulated only 50% compared with that against WT strain in the 2-dose or 3-dose vaccination of SYS6006 in NHPs. It suggested that SYS6006 kept relatively strong neutralization capability against Omicron variants. The 3^rd^ dose of vaccination in NHPs could significantly enhance the neutralization titers against Delta and Omicron BA.2 variants, which was consistent with previous study (12, 18). The antibody titers of SYS6006 against Delta variant maintained high levels in both mouse and monkey for over 6 months, indicating the persistence of humoral immune protection induced by SYS6006. Our findings were consistent with previous reports demonstrating that the LNP-delivering mRNA vaccine or modified vaccinia virus ankara (MVA) vaccine expressing spike antigen could induce robust and prolonged germinal center reactions, recruiting memory B cells as well as new clones for the durable protective antibody responses against SARS-CoV-2 virus (9, 19).

The activation of antigen-specific Tfh cell supported long-term immune response of B cell, and IL21 was the canonical cytokine produced by Tfh cells (20). *In vitro* antigen stimulation and FACS analysis showed that over 5% Tfh cell were activated for IL-21 responses against S protein of SARS-CoV-2 in NHPs with SYS6006 immunization, even higher than the ratio of Tfh activation induced by mRNA-1673 from Moderna (14). This result confirmed that SYS6006 was able to induce long-term B cell response. The induction of CD4^+^ type 2 helper T-cell (Th2) (IL4, IL5, or IL13) responses has been associated with vaccine-associated enhanced respiratory disease (VAERD), as seen in some patients with respiratory virus infections (21). The Th2 cell response was not affected by SYS6006 immunization, which suggested a good safety profile of SYS6006 (22).

Because of biosafety facility limitations, we are not able to obtain protection efficacy data in mouse or NHP under the infection of prevalent SARS-CoV-2 variant such as Omicron BA.2 or BA.5.According to previous report, the mRNA vaccines expressing spike antigens such as mRNA-1273 and BNT162b could induce neutralizing immunity against Omicron variant in boost immunization, only 4-6-fold lower than the immunity against wild type strain (23). Based on the comparison with neutralizing antibody levels in macaques vaccinated with BNT162b2, protective immunity against Omicron variant can be expected in most macaques immunized with three doses of SYS6006 (12). A previous study demonstrated that the neutralizing antibody titer against the Omicron variant 6 months after the third dose of mRNA-1273 was 6.3-fold lower than that of one month after the boost vaccination (24). Thus, understanding the dynamic changes of SYS6006-mediated antibodies is still necessary.

In conclusion, the present study demonstrated the high immunogenicity and protection efficacy of SYS6006 against SARS-CoV-2 in multiple animal models. The strong neutralization effects of SYS6006 and other mRNA vaccines against Delta and Omicron variants highlight the power of mRNA vaccines in the protection against the frequently-varied SARS-CoV-2 virus.

## Data availability statement

The raw data supporting the conclusions of this article will be made available by the authors, without undue reservation.

## Ethics statement

The animal study was reviewed and approved by Pharmaron TSP IACUC (IACUC No.: 21-375), and IACUC at JOINN Laboratories (IACUC serial number: ACU21-1700).

## Author contributions

KX, HY, YW, MD and GW were involved in the study design and supervision. WL, YL, LL, YS, JZ and MY were involved in data acquisition, analysis, and interpretation. BK and XW drafted the manuscript. All authors were involved in critical revision of manuscript and approved the submitted version.

## Supplementary material

Supplementary Figure 1 The GMTs of S1-specific IgG antibodies against Delta variant in the sera of monkeys under the immunization of 30 μg SYS6006 and a 21-day interval. Cynomolgus macaques were intramuscularly immunized with 30 μg SYS6006 and boosted once with a 21-day interval. Sera were collected at the indicated time points and used to detect the GMTs of S1-specific IgG antibodies against Delta variant. Data are presented as geometric mean ± geometric SD. GMT, geometric mean titer.

Supplementary Figure 2 The activity of memory B cells induced by SYS6006 in mice. 6-8-week-old female BALB/c mice were immunized intramuscularly twice with the indicated doses of SYS6006 with a 21-day interval. The splenocytes were collected 7 days after the 2^nd^ vaccination, expanded with R848 and IL2 for 4 days, stimulated with RBD **(A)** and spike **(B)** antigens, and tested for B cell activity *via* mouse IgG ELISpot assay (n=6). Data are presented as mean ± SD. RBD, receptor-binding domain.

Supplementary Figure 3 Gating strategy for analysis of IL21^+^ Tfh cells. The peripheral blood mononuclear cells collected from immunized NHPs shown in were stained with L/D FVS700, CD3 APC-Cy7, CD8 BV480, CD45RA PE-Cy5, CCR7 BV421, CXCR5 PE, PD-1 BV785, ICOS PE-Cy7and IL21 APC, and tested for the rates of IL21^+^ Tfh cells *via* FACS. Tfh cells were gated as singlets, living, CD3^+^, CD8^-^, CCR7^+^, CD45RA^-^, CXCR5^+^, PD-1^+^ and ICOS^+^ lymphocytes. One case immunized with 30μg SYS6006 was presented as a gating example. NHPs, non-human primates; Tfh, T follicular helper cell; PMA, phorbol 12-myristate 13-acetate.

Supplementary Figure 4 Gating strategies for analysis of IFNγ^+^ and IL2^+^ Th1 cells and IL4^+^ and IL13^+^ Th2 cells. The peripheral blood mononuclear cells collected from immunized NHPs were stained with L/D FVS660, CD3 APC-Cy7, CD8 BV480, CD4 PerCP, IL2 BV750, IFNγ Alexa Fluro700**,**IL4 FITC and IL13 BV421, and tested for the rates of Th1 and Th2 cells *via* FACS. One case immunized with 30μg SYS6006 was presented as a gating example.

Supplementary Figure 5 The antigen-specific Th1 cells in NHPs immunized with two different doses of SYS6006. Cynomolgus macaques were intramuscularly immunized with the indicated doses of SYS6006 and boosted with a 21-day interval using the same dose. The peripheral blood mononuclear cells were collected from NHPs 50 days after the 1^st^ vaccination, stimulated with a peptide pool of SARS-CoV-2 S protein, and tested for the rates of IL2^+^ **(A)** and IFNγ^+^ **(B)** Th1 cells *via* FACS analysis (n=2). Data are presented as mean ± SD. PMA/Ionomycin was positive control to stimulate T cells. NHPs, non-human primates; PMA, phorbol 12-myristate 13-acetate.
