# Supplementary figures and images for: A novel mRNA vaccine, SYS6006, against SARS-CoV-2
Source: Front Immunol. 2023 Jan 5;13:1051576. doi: 10.3389/fimmu.2022.1051576 (PMC9849951; doi:10.3389/fimmu.2022.1051576)

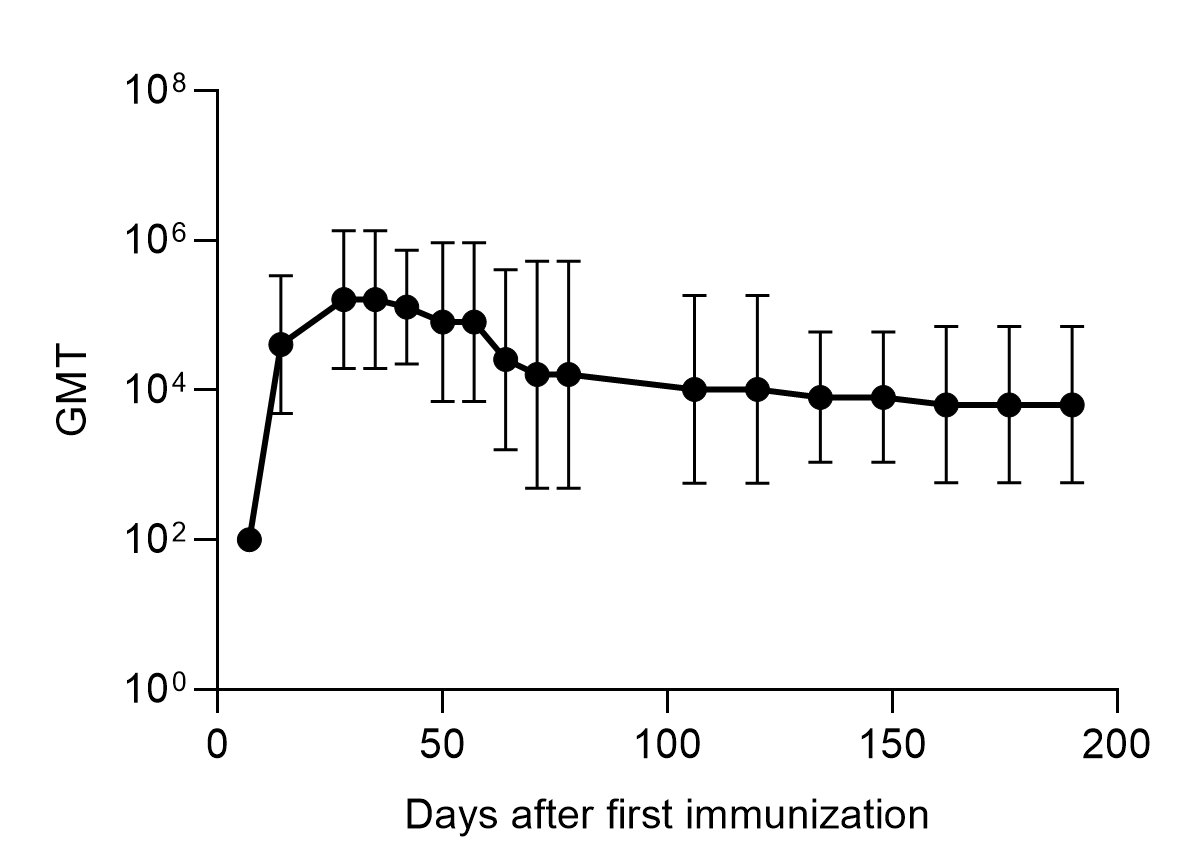

Supplement: Supplementary Figure 1 — The GMTs of S1-specific IgG antibodies against Delta variant in the sera of monkeys under the immunization of 30 μg SYS6006 and a 21-day interval. Cynomolgus macaques were intramuscularly immunized with 30 μg SYS6006 and boosted once with a 21-day interval. Sera were collected at the indicated time points and used to detect the GMTs of S1-specific IgG antibodies against Delta variant. Data are presented as geometric mean ± geometric SD. GMT, geometric mean titer. [file Image_1.tif]

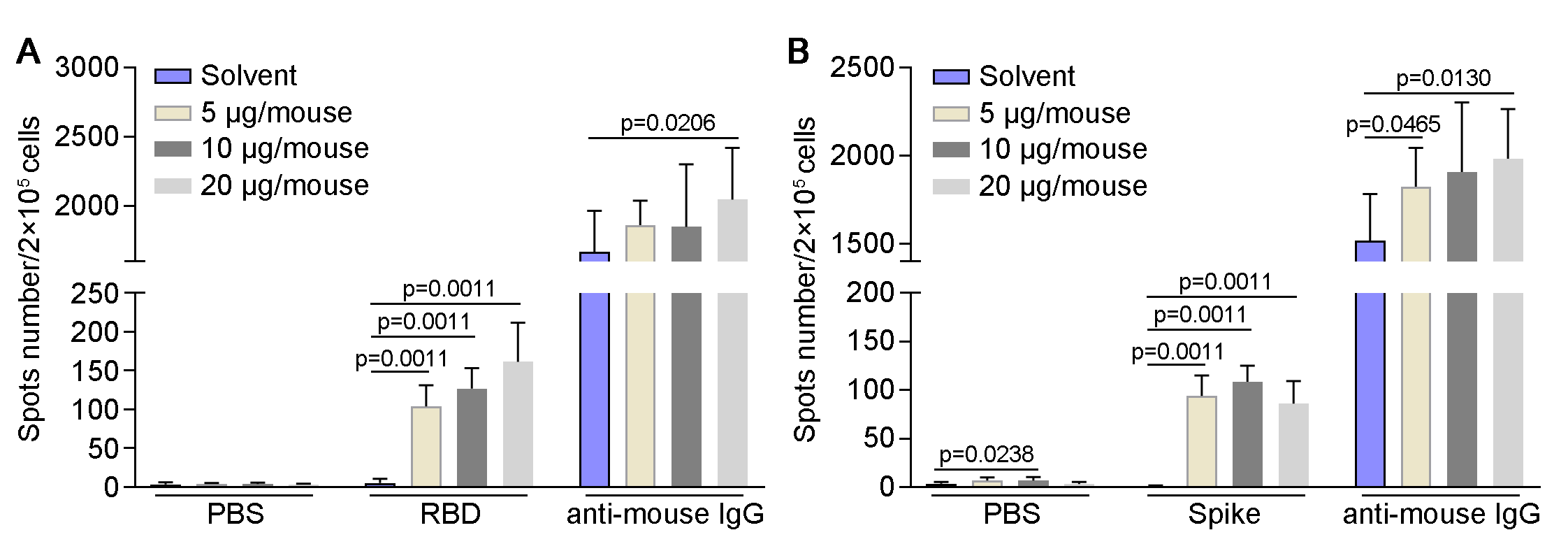

Supplement: Supplementary Figure 2 — The activity of memory B cells induced by SYS6006 in mice. 6-8-week-old female BALB/c mice were immunized intramuscularly twice with the indicated doses of SYS6006 with a 21-day interval. The splenocytes were collected 7 days after the 2nd vaccination, expanded with R848 and IL2 for 4 days, stimulated with RBD (A) and spike (B) antigens, and tested for B cell activity via mouse IgG ELISpot assay (n=6). Data are presented as mean ± SD. RBD, receptor-binding domain. [file Image_2.tif]

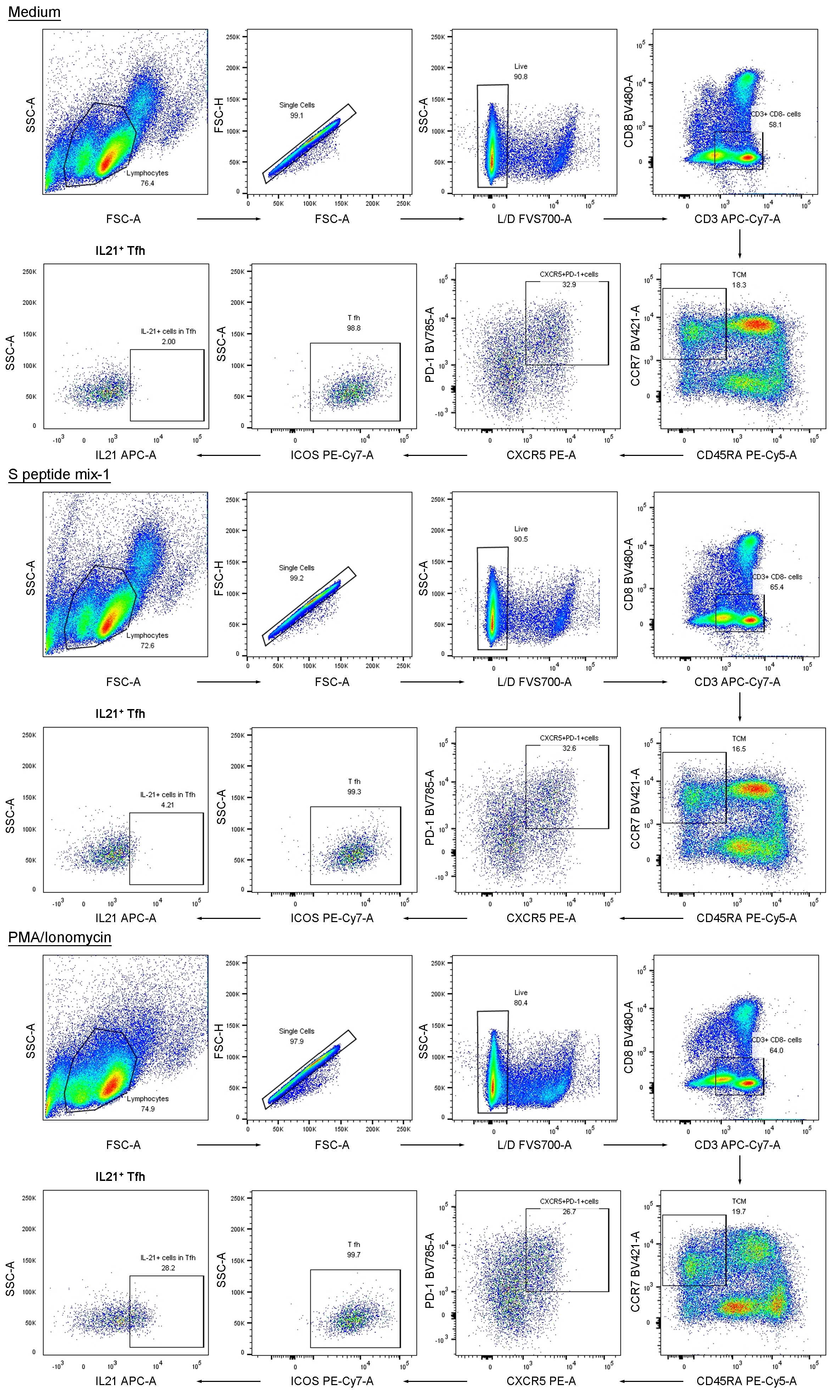

Supplement: Supplementary Figure 3 — Gating strategy for analysis of IL21+ Tfh cells. The peripheral blood mononuclear cells collected from immunized NHPs shown in were stained with L/D FVS700, CD3 APC-Cy7, CD8 BV480, CD45RA PE-Cy5, CCR7 BV421, CXCR5 PE, PD-1 BV785, ICOS PE-Cy7and IL21 APC, and tested for the rates of IL21+ Tfh cells via FACS. Tfh cells were gated as singlets, living, CD3+, CD8-, CCR7+, CD45RA-, CXCR5+, PD-1+ and ICOS+ lymphocytes. One case immunized with 30μg SYS6006 was presented as a gating example. NHPs, non-human primates; Tfh, T follicular helper cell; PMA, phorbol 12-myristate 13-acetate. [file Image_3.tif]

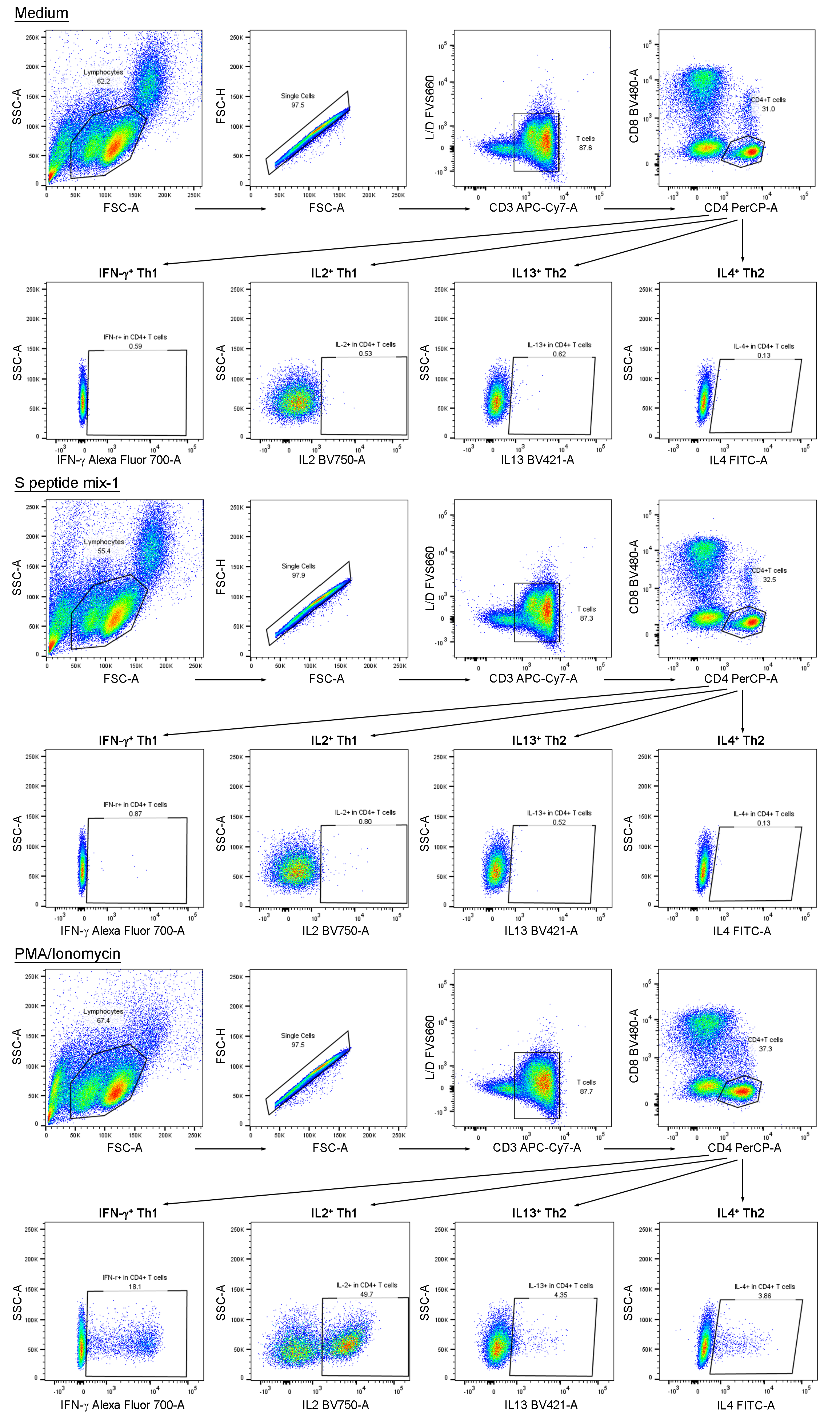

Supplement: Supplementary Figure 4 — Gating strategies for analysis of IFNγ+ and IL2+ Th1 cells and IL4+ and IL13+ Th2 cells. The peripheral blood mononuclear cells collected from immunized NHPs were stained with L/D FVS660, CD3 APC-Cy7, CD8 BV480, CD4 PerCP, IL2 BV750, IFNγ Alexa Fluro700,IL4 FITC and IL13 BV421, and tested for the rates of Th1 and Th2 cells via FACS. One case immunized with 30μg SYS6006 was presented as a gating example. [file Image_4.tif]

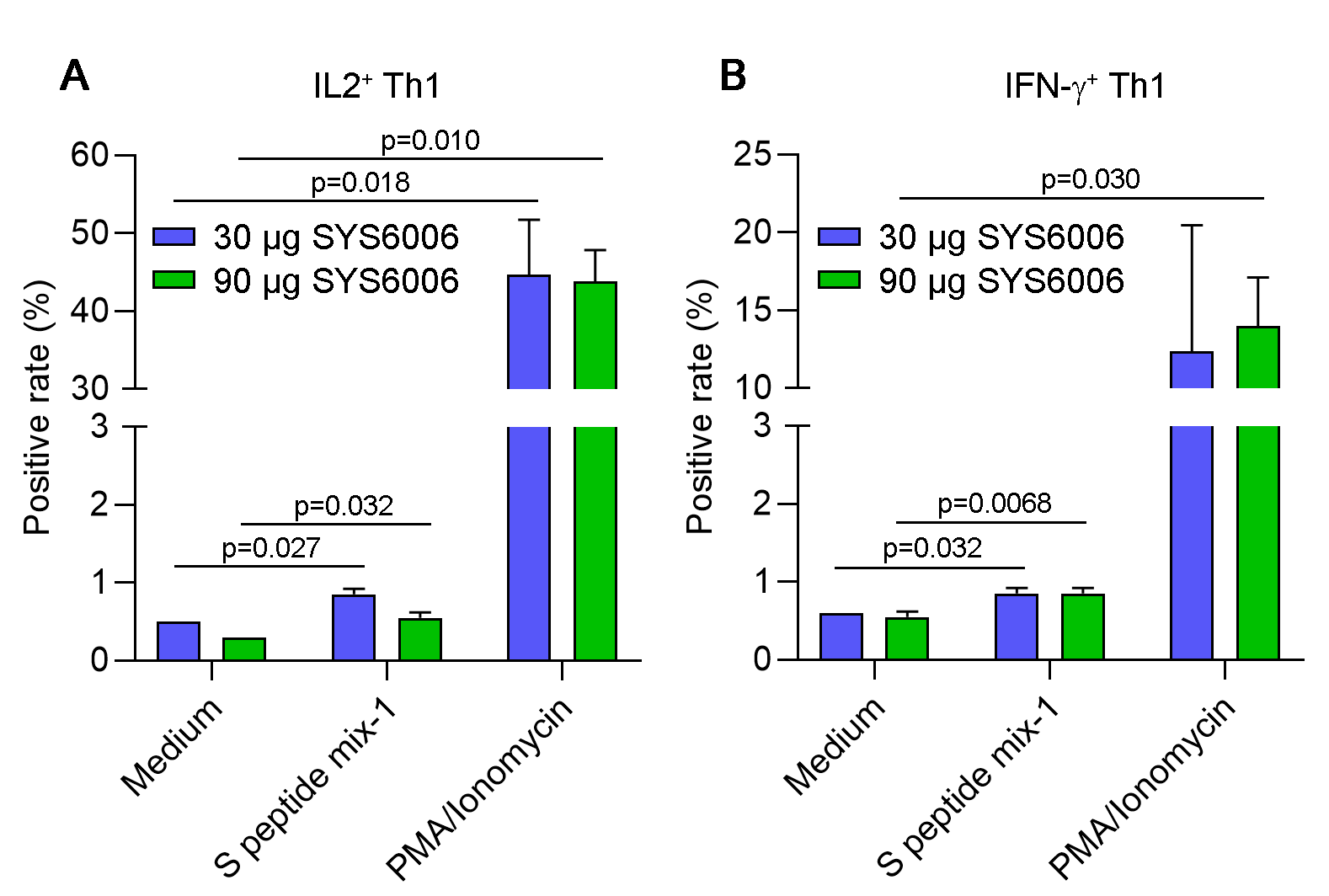

Supplement: Supplementary Figure 5 — The antigen-specific Th1 cells in NHPs immunized with two different doses of SYS6006. Cynomolgus macaques were intramuscularly immunized with the indicated doses of SYS6006 and boosted with a 21-day interval using the same dose. The peripheral blood mononuclear cells were collected from NHPs 50 days after the 1st vaccination, stimulated with a peptide pool of SARS-CoV-2 S protein, and tested for the rates of IL2+ (A) and IFNγ+ (B) Th1 cells via FACS analysis (n=2). Data are presented as mean ± SD. PMA/Ionomycin was positive control to stimulate T cells. NHPs, non-human primates; PMA, phorbol 12-myristate 13-acetate. [file Image_5.tif]
